# Supplementary material for: Management of childhood and adolescent latent tuberculous infection (LTBI) in Germany, Austria and Switzerland
Source: PLoS One. 2021 May 10;16(5):e0250387. doi: 10.1371/journal.pone.0250387 (PMC8109774; doi:10.1371/journal.pone.0250387)
Supplement: S1 Appendix — (PDF) [file pone.0250387.s001.pdf]

S1 Appendix: **Survey: Management of childhood and adolescent latent tuberculous infection (LTBI) in Germany, Austria and Switzerland**

English version

	Chapter	Question	Answer options
1	Details of participating centre		
1.1		Your institution:	
			University hospita
			Central hospital/T
			District general ho
			Private hospital
			Private Practice
			Shared practice or
			Public Health Serv Centre
1.2		Country:	
			Germany
			Austria
			Switzerland
1.3		Federal state (Germany):	
			Baden-Württembe
			Bavaria
			Berlin
			Brandenburg
			Bremen
			Hamburg
			Hesse
			Mecklenburg-Vor
			Lower Saxony
			North Rhine-West

			Rhine Land-Palati
			Saarland
			Saxony
			Saxony-Anhalt
			Schleswig-Holstei
			Thuringia
		Federal state (Austria):	
			Burgenland
			Carinthia
			Lower Austria
			Salzburg
			Styria
			Tyrol
			Upper Austria
			Vienna
			Vorarlberg
		Canton (Switzerland):	
			Aargau
			Appenzell-Ausser
			Appenzell-Innerrh
			Basel-Landschaft
			Basel-Stadt
			Bern
			Fribourg
			Geneva
			Glarus
			Grisons
			Jura
			Luzern
			Neuchâtel
			Nidwalden
			Obwalden

			Schaffhausen
			Schwyz
			Solothurn
			St. Gallen
			Thurgau
			Ticino
			Uri
			Valais
			Vaud
			Zug
			Zürich
1.4		Your professional position?	
			Senior physician/I
			Specialist (i.e. ID,
			Resident physiciar
			Public health offic
			Other:
1.5		How many paediatric / adolescent TB cases (disease, LTBI, exposure) do you manage every year approximately?	
			<10
			10-50
			50-200
			>200
1.6		How often is an index patient or an exposition to a person infected with TB known to you in suspected TB cases?	
			Always (<90% of
			>50-90% of cases
			<50% of cases
1.7		How often do you retrieve information (country of origin, infectiousness, resistance	

		profile of the germ) about the most likely index case?	
			Always (<90% of
			>50-90% of cases
			<50% of cases
2	What resources do you routinely use for Mycobacterium tuberculosis TB diagnostics in children and young people aged 0 to 15 years at your institution?		
2.1		Tuberculin Skin Test (TST)?	
			Yes
			No
2.2		Interferon Gamma Release Assays (IGRA)?	
			Yes
			No
2.3		If yes (2.2), which IGRA do you use?	
			Quantiferon-GOL (Qiagen)
			T-Spot TB (Oxfor
			Both
2.4		Has the recent shortage of PPD/RT23 had an impact on your ability to care for children with TB / LTBI?	
			Yes
			No
2.5		If yes (2.4), did you use another tuberculin preparation instead?	
			No
			Yes
			If yes, which one?
3	Which approach do you routinely use to exclude active tuberculosis?		
3.1		Radiology/Imaging Techniques:	
3.1.1			Chest X-Ray (CX
			Yes

			No
3.1.2			Chest CT scan?
			Yes
			No
3.1.3			Ultrasound/Sonog
			Yes
			No
3.2		Microbiology:	
3.2.1			Sputum / Gastric a
			Yes
			No
3.2.2			Sputum / Gastrid a
			Yes
			No
3.2.3			PCR-based techni / appropriate mate (Cepheid)?
			Yes
			No
4	Aspects of LTBI diagnosis		
4.1		Your diagnostic work up for if LTBI is suspected due to patient history and/or exposure	
			TST alone
			TST + CXR perfo
			IGRA alone
			IGRA + CXR per
			TST + IGRA
			TST + IGRA + C
			CXR only if TST
			Unknown
			Other:
4.2		Do you use a different approach for **children < 5 years**?	

			Yes
			No
			Unknown
4.3		If yes (4.2):	
			TST alone
			TST + CXR perfo
			IGRA alone
			IGRA + CXR per
			TST + IGRA
			TST + IGRA + C
			CXR only if TST
			Unknown
			Other:
5	Preventive chemotherapy of LTBI		
5.1		What is your first line preventive therapy in case of an index case with drug susceptible TB or missing anamnestic hints pointing at exposure to drug resistant TB?	
			Isoniazid (INH) a
			Rifampicin (RMP
			INH/RMP combi
			Other:
			Unknown
5.2		For how long do you treat a child with INH monotherapy?	
			3 months
			4 months
			6 months
			9 months
			Other:
			Unknown / Not ap
5.3		For how long do you treat a child with RMP monotherapy?	
			3 months

			4 months
			6 months
			9 months
			Other:
			Unknown / Not ap[illegible]
5.4		For how long do you treat a child with INH/RMP combination therapy?	
			3 months
			4 months
			6 months
			9 months
			Other:
			Unknown / Not ap[illegible]
5.5		Frequency of dosing of anti-TB agents:	
			Daily
			2 x /week
			3 x / week
			Other:
5.6		What dose do you use for INH?	
			~5 mg/kg/day (3-[illegible]
			~10 mg/kg/day (7[illegible]
			~15 mg/kg/day (>[illegible]
			Other:
			_____ mg/kg/day [illegible] application
			_____ mg/kg/day [illegible] application
5.7		What dose do you use for RMP?	
			~10 mg/kg/day
			~15 mg/kg/day
			>15 mg/kg/day
			Other:

			_____ mg/kg/day application
			_____ mg/kg/day application
5.8		Do you usually conduct vitamin B6 substitution therapy in case of INH treatment?	
			Yes
			No
			Yes, under the fol
5.9		What **laboratory** parameters do you routinely screen for in children **before starting** preventive therapy (multiple answers possible)?	
			Full blood count
			LFTs (ALAT, AS
			Electrolytes (Na^+,
			Creatinine
			CRP
			Additional blood t
			None
5.10		What **laboratory** parameters do you routinely screen for in children **during** preventive therapy (multiple answers possible)?	
			Full blood count
			LFTs (ALAT, AS
			Electrolytes (Na^+,
			Creatinine
			CRP
			Additional blood t
			None
5.11		How often do you conduct routine clinical follow-ups during preventive therapy (initial presentation excluded)?	
			There is no routin
			1x at the end of tre
			1-2x during therap

			3-4x during therap
			>4x during therap
			Unknown
5.12		How do you monitor compliance during treatment phase? (Multiple answers possible)	
			Not at all
			Interrogation
			Check number of
			Routine urine sam
			Routine urine sam (INH, RMP)
			Drug levels in sali
			Plasma / serum dr
			Unknown
			Other:
5.13		Do you always take a chest X-ray at end of treatment?	
			Yes
			No
			Unknown
5.14		Do you routinely see patients again for follow-up after end of treatment?	
			Yes
			No
			Unknown
5.15		If yes (5.14), when does this routine follow-up take place? (multiple answers possible)	
			6 months after enc
			12 months after er
			24 months after er
			________ after en
5.16		When would you start chemoprophylaxis in a child exposed to a case of multi-drug-resistant TB (MDR-TB) and not showing any clinical signs of infection or active disease (TST and	

		IGRA negative), provided a drug-sensitivity testing from presumed index case is available?	
			Always
			Always in childre[illegible]
			Only when the ris[illegible] (close contact or h[illegible] patient)
			Only when the ris[illegible] a child younger th[illegible]
			Unknown
			Never (Watch & [illegible]
			I always consult o[illegible]
5.17		When do you start chemoprophylaxis according to antibiotic resistance profile in a child exposed to multi-drug-resistant TB (MDR-TB) and now showing signs of infection?	
			Always
			Always in childre[illegible]
			Individual decisio[illegible] parents´ desire for[illegible] medication, etc.)
			Unknown
			Never (Watch & [illegible]
			I always consult o[illegible]
6	Specific questions on individual institution setting		
6.1		How would you estimate the percentage of patients presenting for presumed TB (active disease, LTBI, exposition) being of migrant background?	
			0
			0 - <20%
			20 - <50%
			50 - <80%
			>80%

6.2		Are there any further educational / teaching events on pediatric TB or LTBI close to where you work?	
			Yes
			No
			Unknown
6.3		Do you also care for patients with relevant comorbidities (HIV, patients on biologicals, immune deficiencies)?	
			Yes
			If yes, which ones
			No
6.4		How often do your patients started on preventive chemotherapy for LTBI finish the complete treatment course?	
			Always (>90%)
			Very often (70-90
			Often (50-70%)
			Rarely (<50%)
6.5		To what extent do you take into account the social (family, friends) or further environmental factors of a patient (more than one answer possible)?	
			Seeing and exami
			No individual app information on ris given to close con
			No special focus c
6.6		What guidelines do you turn to for advice on LTBI management in children and adolescents (more than one answer is possible)?	
			DZK „New Recor environmental exa tuberculosis“ (201
			DZK „Recommen chemotherapy and tuberculosis in adu

			DGPI handbook
			TB handbook (CH[illegible]
			International / Eu[illegible]
			Other:
			Unknown
