# Supplementary material for: Management of childhood and adolescent latent tuberculous infection (LTBI) in Germany, Austria and Switzerland
Source: PLoS One. 2021 May 10;16(5):e0250387. doi: 10.1371/journal.pone.0250387 (PMC8109774; doi:10.1371/journal.pone.0250387)
Supplement: S2 Appendix — (PDF) [file pone.0250387.s002.pdf]

S2 Appendix: Availability of training on childhood TB

Eighty-three/173 (48.0%) respondents stated to have access to regular training or educational courses on childhood TB, 43/173 (24.9%) stated to not have access and 47/173 (27.2 %) did not know or did not respond.

Continuous educational training is required in order to enhance awareness and knowledge on the management of paediatric LTBI.
